# Evolution of the Arabidopsis telomerase RNA

**DOI:** 10.3389/fgene.2012.00188

**Published:** 2012-09-24

**Authors:** Mark A. Beilstein, Amy E. Brinegar, Dorothy E. Shippen

**Affiliations:** ^1^School of Plant Sciences, University of ArizonaTucson, AZ, USA; ^2^Department of Biochemistry and Biophysics, Texas A&M University, College StationTX, USA

**Keywords:** telomerase, TER, gene duplication, Brassicaceae, phylogenetics

## Abstract

The telomerase reverse transcriptase promotes genome integrity by continually synthesizing a short telomere repeat sequence on chromosome ends. Telomerase is a ribonucleoprotein complex whose integral RNA subunit TER contains a template domain with a sequence complementary to the telomere repeat that is reiteratively copied by the catalytic subunit. Although TER harbors well-conserved secondary structure elements, its nucleotide sequence is highly divergent, even among closely related organisms. Thus, it has been extremely challenging to identify TER orthologs by bioinformatics strategies. Recently, TER was reported in the flowering plant, *Arabidopsis thaliana*. In contrast to other model organisms, *A. thaliana* encodes two TER subunits, only one of which is required to maintain telomere tracts *in vivo*. Here we investigate the evolution of the loci that encode TER in Arabidopsis by comparison to the same locus in its close relatives. We employ a combination of PCR and bioinformatics approaches to identify putative TER loci based on syntenic regions flanking the TER1 and TER2 loci of *A. thaliana*. Unexpectedly, we discovered that the genomic regions encoding the two *A. thaliana* TERs occur as a single locus in other Brassicaceae. Moreover, we find striking sequence divergence within the telomere template domain of putative TERs from Brassicaceae, including some orthologous loci that completely lack a template domain. Finally, evolution of the locus is characterized by lineage-specific events rather than changes shared among closely related species. We conclude that the Arabidopsis TER duplication occurred very recently, and further that changes at this locus in other Brassicaceae indicate the process of TER evolution may be different in plants compared with vertebrates and yeast.

## INTRODUCTION

The ends of eukaryotic chromosomes are typically composed of simple arrays of G-rich repeats. In plants, this sequence is TTTAGGG, while vertebrate telomeres consist of the related sequence TTAGGG. Telomeric DNA is synthesized and maintained through the action of the telomerase reverse transcriptase. Telomerase is a ribonucleoprotein (RNP) enzyme minimally comprised of a reverse transcriptase subunit (TERT) responsible for catalytic activity along with an RNA moiety, the telomerase RNA (TER). TER contains a short nucleotide sequence complementary to the G-rich strand of telomeric DNA. This region is termed the template domain, and it is reiteratively copied by TERT during the synthesis of telomeric repeats.

The discovery of TER molecules from vertebrates, budding yeast, and ciliates has revealed conserved structural elements essential for the function of the telomerase RNP. In addition to the template domain, TER molecules contain a central core composed of a pseudo-knot and template boundary region (Chen and Greider, 2004; Lin et al., 2004). TER also acts as a scaffold to which other non-catalytic telomerase-associated proteins bind (Zappulla and Cech, 2004). Such factors facilitate the assembly and proper localization of the RNP and its recruitment to the chromosome terminus (Autexier and Lue, 2006).

Despite the conservation of structural elements, TER is extremely divergent at the nucleotide level, making bioinformatics approaches based on sequence similarity of the TER locus difficult, even among closely related species. Notably, TER from fission yeast was uncovered only 4 years ago using a biochemical approach (Leonardi et al., 2008; Webb and Zakian, 2008), while the *Caenorhabditis elegans* TER has yet to be discovered. TER from the flowering plant *Arabidopsis thaliana* was reported in 2011, following biochemical purification of the plant enzyme (Cifuentes-Rojas et al., 2011). Remarkably, Arabidopsis encodes two TER molecules (TER1 and TER2), a situation unique among all species from which TER has been isolated. Both Arabidopsis TERs have a 10-nucleotide (nt) template region (5′-CUAAACCCUA-3′) complementary to one and a half copies of the TTTAGGG telomeric DNA repeat. In addition, both of these RNAs assemble with TERT *in vitro*, to reconstitute an enzymatically active telomerase particle. However, only TER1 is required for telomere maintenance *in vivo*, leaving the function of TER2 unresolved (Cifuentes-Rojas et al., 2011).

The TER1 locus is found on chromosome one. It partly overlaps the coding region of the recently characterized RAD52 homolog in Arabidopsis (Samach et al., 2011), and encodes a 748 nt RNA with a 235 nt 5′ region highly divergent at the nucleotide level from TER2. The TER2 locus is on chromosome five, lies partly within the 5′ UTR of the uncharacterized open reading frame AT5G24670 (designated TRNA ADENOSINE DEAMINASE 3 -TAD3), but in the opposite orientation, and encodes a 784 nt RNA. TER1 and TER2 share a 220 nt stretch with ~90% sequence identity. In TER2, a 529 nt sequence highly divergent from TER1 splits the conserved stretch into two blocks: conserved region 1 (CR1) 114 nt and conserved region 2 (CR2) 96 nt (Cifuentes-Rojas et al., 2011). The pattern of sequence similarity and divergence between the two TERs precludes analyses of compensatory base changes to elucidate TER secondary structure; the aligned molecules either lack sufficient changes in regions of similarity, or cannot be confidently aligned in regions of divergence.

Our goal was to identify the TER loci from close relatives of Arabidopsis to explore the evolutionary relationship between these two genes and, using phylogeny, to develop a model of TER secondary structure in the plant kingdom. To obtain sequence data sufficient for such an analysis, we took advantage of highly conserved protein coding regions flanking the TER loci of Arabidopsis to identify homologous regions in closely related species in the plant family Brassicaceae. For these studies, we employed a combination of PCR and bioinformatics approaches similar to those employed by Dandjinou et al. (2004) in their analysis of TER phylogeny within the *sensu stricto* group of *Saccharomyces*. Because the TER locus is duplicated in Arabidopsis, our sampling of species was also designed to determine where in the evolutionary history of the family this duplication occurred.

## MATERIALS AND METHODS

Three different strategies were used to identify TER loci from species in the plant family Brassicaceae. The family comprises three major lineages (I–III; Beilstein et al., 2006, 2008) and the genus *Aethionema*, an early diverging group sister to the rest of Brassicaceae. Sampled species represent two of the three phylogenetic lineages with denser sampling around *Arabidopsis thaliana* (lineage I) to ensure identification of the branch along which the TER duplication occurred. In the initial phases of the project, genomic data were obtained from the U.S. Department of Energy Joint Genomes Institute (JGI) for *A. lyrata*, and from *Brassica rapa* bacterial artificial chromosomes (BACs) in GenBank. To obtain putative TER loci from sampled species lacking genomic resources, a combination of hiTAIL PCR (Liu and Chen, 2007) and standard PCR were used to amplify putative TER loci from genomic DNA. In later phases of the project, we mined additional genomic data that became available through www.phytozome.net, JGI’s web portal for plant genomic data.

### BIOINFORMATICS

To obtain putative TER loci from species for which whole genome sequence was available, a bioinformatics approach was employed. BLAST searches were queried with the portion of the *A. thaliana* RAD52 cDNA that overlaps the TER1 locus and the first 100 base pairs (bp) of the TAD3 predicted cDNA adjacent to the TER2 locus. Nucleotide BLAST searches against the *A. lyrata* whole genome sequence available through the JGI web portal were performed with default settings to identify orthologs of both genes. Using GenBank, we queried BAC sequences in the nucleotide collection specifying *B. rapa *as the target organism. When the *Schrenkiella parvula* (*Eutrema parvulum*) genome sequence was completed and deposited on GenBank, nucleotide BLAST was performed against its genome using the same strategy. Reciprocal nucleotide BLASTs were performed for all returned sequences to determine orthology with the query sequences.

Nucleotide BLAST searches against *Brassica rapa*, *Capsella rubella*, and *Eutrema salsugineum* (formerly *Thellungiella halophila*) whole genome sequences employed the standard settings in Phytozome. Searches were performed as described above, using RAD52 or TAD3 cDNA as query, and reciprocal BLASTs were performed on all returned sequences. In addition to these publicly available data, additional BLAST results were provided from the emerging genome sequence of *Aethionema arabicum* pre-publication (http://vegi.cs.mcgill.ca/).

### PCR SCREENS

To obtain putative TER loci from species lacking whole genome data, a degenerate PCR approach was employed. Primer design took advantage of the fact that coding regions flanking the two TER loci in *A. thaliana *are relatively well conserved. First we identified the RAD52 and TAD3 orthologs from *A. lyrata* and *B. rapa* using available genomic resources and aligned each gene with its respective *A. thaliana* copy. Using highly conserved coding regions within these genes, a series of modestly degenerate primers were designed for hiTAIL PCR in PrimaClade (Gadberry et al., 2005), and subsequently amplified regions adjacent to each gene for 10 species lacking genomic data: *A. arenosa*, *A. neglecta*, *Boechera platysperma*, *Cardamine hirsuta*, *Cardamine rhomboidea*, *Crucihimalaya lasiocarpa*, *Dimorphocarpa wislizenii*, *Lepidium draba*, *Olimarabidopsis pumila*, *Turritis glabra*. In each case, all robustly amplified bands >150 bp produced during hiTAIL PCR were gel extracted, cloned (TOPO-TA, Invitrogen Inc.), and sequenced. A minimum of two clones were sequenced for each PCR fragment to increase the chances of finding alternative loci. For each species resulting sequence data were used in BLAST searches against the *A. thaliana* genome to determine orthology of the coding portions of the amplified fragments. hiTAIL PCR reactions amplified a single locus in all species (see results) regardless of the band sequenced. Sequences were aligned, and degenerate primers were used to amplify the entire locus using standard PCR methods. Degenerate primers for standard PCR were placed in the second exon of RAD52 and the third exon of TAD3.

### ALIGNMENT AND EVOLUTIONARY INFERENCE

The PCR and BLAST screens were designed to return sequences orthologous to RAD52 and TAD3, along with adjacent sequence, from all sampled species. The sequences were aligned to determine whether adjacent regions contained putative TER loci. First, the RAD52 and TAD3 orthologous coding regions were aligned by eye from all sampled species with the *A. thaliana* genes using the visual alignment interface of the computer software MacClade (Maddison and Maddison, 2005). With these genes as anchors, the regions adjacent to them were aligned. The TER telomere template domain served as a landmark to aid alignment. For species lacking a template, the alignment program MUSCLE (Edgar, 2004) was used with default parameter settings to test if sequence similarity not evident under manual alignment was present.

To determine the evolutionary history of the TER duplication, we inferred a maximum likelihood phylogeny of all the recovered loci and TER1 and TER2 from Arabidopsis. Likelihood searches were performed in RAxML version 7.2.3 (Stamatakis, 2006) using the GTRGAMMA option from an alignment of 330 nt. Support for nodes in the tree was assessed using 100 bootstrap replicates and implemented in RAxML. The gene tree we recovered agreed with the accepted organismal phylogeny of the family (Beilstein et al., 2006, 2008), and thus we integrated the duplication history inferred from this gene tree with the organismal phylogeny. To test scenarios of evolution, we used the alignment of all 16 loci in the program MacClade, which reconstructs the evolution of each site in the alignment under a parsimony framework by inferring ancestral character states throughout a given tree. For these analyses, we chose the well-resolved organismal tree, which did not differ significantly from the gene tree.

## RESULTS

We obtained putative TER loci from 14 species in the family Brassicaceae using a combination of PCR and bioinformatics approaches. These approaches were reciprocally illuminating in regard to the recovery of the putative TER loci. For example, BLAST results in *A. lyrata* and *B. rapa* were used to design primers for hiTAIL PCR. Results from early rounds of hiTAIL PCR indicated that the RAD52 and TAD3 genes might be located on the same chromosome in some species of Brassicaceae. Similarly, BLAST results returned the same general genomic location for both RAD52 and TAD3. Putative TER loci recovered using PCR approaches were submitted to GenBank (*A. arenosa *JX546281; *A. neglecta *JX546280; *Aethionema arabicum *JX546289; *Camelina hispida *JX546282; *Cardamine hirsuta *JX546284; *C. rhomboidea *JX546285; *Crucihimalaya lasiocarpa *JX546286; *Dimorphocarpa wislizenii *JX546283; *Lepidium draba *JX546287).

### THE *A. THALIANA* TER1 AND TER2 LOCI ARE A SINGLE LOCUS IN OTHER BRASSICACEAE

*Arabidopsis thaliana* TER1 and TER2 are encoded on different chromosomes. Thus, our initial bioinformatics screens were designed to identify RAD52 orthologs in *A. lyrata* and *B. rapa*. For both species, a single locus with sequence similarity to *A. thaliana* RAD52 was retrieved; reciprocal BLASTs returned *A. thaliana* RAD52. Next *A. thaliana* TAD3 was queried via BLAST and a single locus with similarity to *A. thaliana* TAD3 was recovered. Surprisingly, these BLAST results also revealed that the two genes are located on the same scaffold in *A. lyrata* and the same BAC in *B. rapa*, although they are encoded on opposite strands. In addition, the start codons are separated by a relatively short intergenic region of 1,711 bp (*A. lyrata*) and 1,120 bp (*B. rapa* ; **Figure 3**).

RAD52 and TAD3 are located on the same assembled genomic scaffold in *Capsella rubella*, *Eutrema salsugineum*, *Schrenkiella parvula*, and *Aethionema arabicum*, whose genome sequences were released during the course of the project or were obtained from collaborators. Sequence data from *A. arabicum* is particularly informative because the genus *Aethionema* is sister to all other Brassicaceae. As for *A. lyrata* and *B. rapa*, BLAST results from these species returned a single locus with sequence similarity to either RAD52 or TAD3. In each case, an intergenic region of varying length separated the two genes: 607 bp (*A. arabicum*), 1567 bp (*C. rubella*), 895 bp (*E. salsugineum*), and 635 bp (*S. parvula*; **Figure 3**).

Both hiTAIL and standard PCR screens indicated that RAD52 and TAD3 are adjacent in *Arabidopsis arenosa*, *A. neglecta*, *Camelina hispida*, *Cardamine hirsuta*, *C. rhomboidea*, *Crucihimalaya lasiocarpa*, *Dimorphocarpa wislizenii*, and *Lepidium draba*. Initial sequence data were generated using hiTAIL PCR, which amplified multiple bands from degenerate primers. Fragments greater than 150 bp were gel-extracted and cloned. However for each species, a single locus with sequence similarity to the target region (either TAD3 or RAD52) was obtained from cloned PCR fragments, regardless of size. In *C. hispida* and *C. lasiocarpa*, hiTAIL PCR products were longer (~2000 bp) than in other species we sampled. Moreover, sequences generated from the cloned *C. hispida* and *C. lasiocarpa* PCR fragments included both the RAD52 and TAD3 orthologs (**Figure 3**). Subsequent PCR amplifications used standard PCR protocols with primers placed in the RAD52 and TAD3 genes. For all species we were able to generate PCR products between these two primers, indicating that the two genes are in the same genomic region (**Figure 3**). Sequence data from these cloned PCR products confirmed the region amplified contained RAD52 and TAD3. The length of the intergenic space for sequenced species was: 587 bp (*A. arenosa*), 538 bp (*A. neglecta*), 597 bp (*C. hirsuta*), 640 bp (*C. rhomboidea*), 564 bp (*Crucihimalaya lasiocarpa*), and 653 bp (*D. wislizenii*). For *C. hispida*, we did not sequence the entire length of the intergenic region, but the sequenced fragment was greater than 550 bp and contained a putative template domain.

In addition to identifying the RAD52 and TAD3 orthologs from the sampled species, we analyzed the sequence of each intergenic region to determine whether it shares greater identity with the *A. thaliana* TER1 or TER2 locus. In nine of the sampled species the majority of the intergenic space shares identity of at least 69% with the 545 bp domain upstream of the TER2 start position (**Figure 3**). In five species, less sequence similarity was detected. In *A. lyrata*, which has the longest intergenic region sampled, 445 bp of the 1711 bp intergenic region shares 77% sequence identity with the TER2 locus, while the 550 bp region upstream of the RAD52 start codon shares 74% sequence identity to a DNA tract located on *A. thaliana* chromosome 2. Roughly 25% (212 bp) of the *E. salsugineum* intergenic region shares 71% identity with TER2. For *C. hispida*, the region 86 bp upstream of the template domain shares 79 % identity with TER2. The *C. rubella* intergenic space shares little identity with TER2, rather a 134 bp segment of its intergenic space shares sequence identity with a DNA tract located several megabases away on *A. thaliana* chromosome 5. Finally, the *A. arabicum* intergenic space shared less than 25% sequence identity with either TER1 or TER2. Thus, the intergenic region in most of the sampled species is more similar to the region 5′ of *A. thaliana* TER2, rather than TER1.

In all species we sampled, RAD52 and TAD3 are located within a segment of 538 to 1711 bp and are encoded on alternate strands. In striking contrast, *A. thaliana* RAD52 and TAD3 are encoded on different chromosomes, and each is adjacent to one of the two TER genes. For the majority of species sampled, the intergenic region is highly similar to the stretch of DNA between the TAD3 start codon and the TER2 start, increasing confidence in the orthology of the region. Integration of this finding with the organismal phylogeny indicates that the event leading to the TER duplication occurred on the branch leading to *A. thaliana* (**Figure 3**), since even other species of the genus have only a single locus with sequence similarity to the two loci in *A. thaliana*. Altogether, these data indicate that the genomic region encoding the two *A. thaliana* TER occurs as a single locus in all other sampled Brassicaceae.

### SEQUENCE DIVERGENCE IN THE TER TEMPLATE DOMAIN WITHIN BRASSICACEAE

**FIGURE 1:**
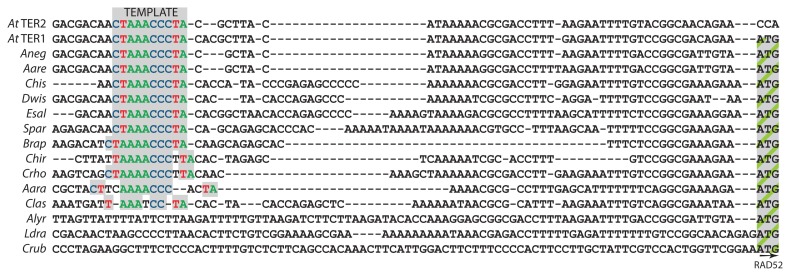
** Retention, degradation, and loss of the template domain within putative Brassicaceae TER genes **.Partial sequence alignment of TER1, TER2 and the corresponding TAD3–RAD52 intergenic region in Brassicaceae species is shown. Sequence similarity through the start codon of RAD52 (far right) is indicated. The colored nucleotides indicate the template domain in *A. thaliana* TER1 and TER2, and the putative template or template-like domains in other species. Species are Aara, *Aethionema arabicum*; At, *Arabidopsis thaliana*; Aare, *A. arenosa*; Aneg, *A. neglecta*; Brap, *Brassica rapa*; Chis, *Camelina hispida*; Chir, *Cardamine hirsuta*; Crho, *C. rhomboidea*; Crub, *Capsella rubella*; Clas, *Crucihimalaya lasiocarpa*; Dwis, *Dimorphocarpa wislizenii*; Esal, *Eutrema salsugineum*; Ldra, *Lepidium draba*; Spar, *Schrenkiella parvula*.

We aligned the TAD3 to RAD52 intergenic regions of the 14 sampled species. Using the alignment, we searched for retention of an *A. thaliana* like template domain upstream of the RAD52 start codon (**Figure 1**). An exact match to the *A. thaliana* template domain was recovered in six species: *A. arenosa*, *A. neglecta*, *C. hispida*, *D. wislizenii*, *E. salsugineum*, and *S. parvula* (**Figures 1** and **3**). Variations on the template domain occurred in five species. Nucleotide changes include the addition of a single extra adenine in *B. rapa*, increasing the stretch of A residues in the putative template domain from three to four nt (**Figure 1**). The same insertion is evident in *C. hirsuta*, *C. rhomboidea*, and *A. arabicum*. The putative template domains of *C. rhomboidea* and *C. hirsuta *are also altered by the insertion of an extra thymine at the 3′ end of the template region. *C. hirsuta* has a substitution of the terminal cytosine to thymine. *Aethionema arabicum* exhibits even more elaborate deterioration of the template domain with a two nt substitution at both the 5′ and 3′ ends. Only the core AAACCC repeat remains intact. *Crucihimalaya lasiocarpa *lacks even this core repeat, with a substitution at one of the central cytosines to thymine. Strikingly, we could find no evidence of a template or template-like domain in the entire length of the intergenic region from three species: *A. lyrata*, *C. rubella*, and *L. draba*. Moreover, no other loci with sequence similarity to the two *A. thaliana* TERs were recovered by BLAST or PCR approaches. We conclude that the template domain within TER is either evolving rapidly, or that alternative TER loci exist for several of the members of the Brassicaceae.

### EVOLUTION OF THE RAD52–TAD3 LOCUS IS CHARACTERIZED BY LINEAGE-SPECIFIC EVENTS

**FIGURE 2:**
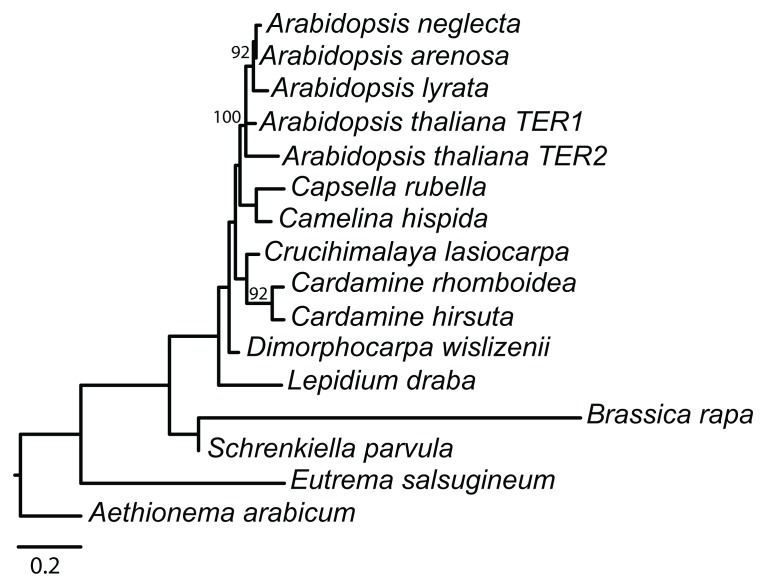
**Phylogeny of 330 nt alignment of Arabidopsis TER1 and TER2 with putative TER loci from 14 other Brassicaceae species.** Values above nodes are from 100 likelihood bootstrap replicates, only values above 60% are reported. Scale bar is 0.2 substitutions/per site.

**FIGURE 3:**
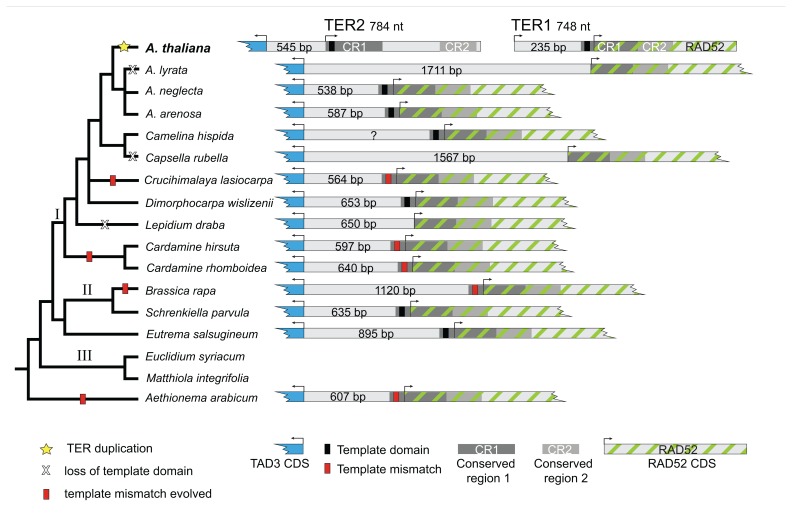
**Phylogeny and organization of the Arabidopsis TER and putative TER loci in Brassicaceae.** *Left*, Brassicaceae tree modified from Beilstein et al. (2010). The most parsimonious reconstruction of the TER locus in Brassicaceae is depicted. Shown are species sampled in this study. Species in lineage III from which TER loci were not obtained are also included for context. Yellow star indicates the duplication event that produced two TER loci in *A. thaliana*. *Right*, schematic diagram of the TAD3–RAD52 locus. *A. thaliana* TER1 and TER2 are encoded on separate chromosomes adjacent to or overlapping these loci. In other Brassicaceae, TAD3 and RAD52 lie on the same chromosome, flanking a single putative TER gene. The region shown is proportional only for the intergenic space. Arrows indicate predicted transcriptional start sites.

Phylogenetic analysis of the TER loci recovered a single likelihood tree (-ln 2564.5971; **Figure 2**). Model parameters from the most likely tree in RAxML included a shape parameter for the γ distribution (α = 1.78325) and a GTR transition matrix (a–c = 0.719862, a–g = 0.697629, a–t = 1.035392, c–g = 0.576520, c–t = 1.201706, g–t = 1.000000). Bootstrap support was generally low, however the *Arabidopsis* clade was resolved with 100% support, and *A. arenosa*, *A. lyrata*, and *A. neglecta* formed a well-supported monophyletic group within the *Arabidopsis* clade (92% bootstrap), indicating that the duplication that produced TER1 and TER2 is restricted to *A. thaliana*. Moreover, the recovered tree is consistent with the accepted organismal phylogeny of the family, and thus we used the organismal tree to infer evolutionary events. Integration of the putative TER loci with the organismal tree revealed that lineage-specific events affecting the locus occurred several times throughout the history of the family. The most parsimonious reconstruction of the evolution of the template indicates that the 5′-CTAAACCCTA-3′ encoding domain is ancestral in Brassicaceae **(Figure 3**). Species in both lineages I and II of the family have the canonical template, indicating they descended from a common ancestor with the template. Data for lineage III are currently missing, despite our efforts to obtain sequences for the group. In *A. arabicum*, there are numerous substitutions in the template domain. However, our data suggest that this is more likely a lineage-specific degradation of the template domain rather than the ancestral condition, especially since such modifications are relatively frequent, occurring in five of the 14 sampled species.

Substitutions in the template domain are independent evolutionary events. For example, insertion of adenine in the A stretch of the template occurred independently at least three times in the family: once each along the branch to *A. arabicum* and *B. rapa*, and once along the branch uniting *C. hirsuta* and *C. rhomboidea*. These latter two species share a thymine insertion at the 3′ end of the template, but *C. hirsuta* also has an independent thymine substitution at the 5′ end. Additional independent lineage-specific substitutions and insertions occurred in *A. arabicum* and *C. lasiocarpa*. Loss of the template domain occurs as an independent lineage-specific event in *A. lyrata*, *C. rubella*, and *L. draba*, since the closest sampled relative of each of these species has the canonical template. Interestingly, loss of the template domain does not appear to be correlated with a general loss of sequence identity in the intergenic region. For example, the *L. draba* intergenic region contains a 287 bp segment with 71% sequence identity to the TER2 locus, and in *A. lyrata* there is a 449 bp segment with 77% identity to TER2. Only in *C. rubella* is there both a loss of the template and a corresponding loss of sequence similarity. Moreover, losses are relatively common, occurring in three of the fourteen species **(Figure 3**). Losses are restricted to lineage I, however our sampling within this lineage is denser than in other groups of the family, suggesting denser sampling might reveal additional losses in other lineages. Finally, duplications of the TER locus are rare; the duplication producing two TER loci occurred along the branch to *A. thaliana *(**Figure 3**).

Interestingly, we found that all of the putative TER loci contain CR1 and CR2 overlapping with the RAD52 gene (**Figure 3**). This configuration is TER1-like in other Brassicaceae, indicating that the 529 nt non-conserved stretch that splits these conserved regions in TER2 is of recent origin. All other sampled species of *Arabidopsis* lack the 529 nt non-conserved stretch, hence the most recent common ancestor of the group is inferred to lack this stretch. The coalescence of *A. thaliana* and other *Arabidopsis* species occurred approximately 13 million years ago (Beilstein et al., 2010), and thus the insertion of this internal non-conserved sequence occurred sometime after this date. Similarly, the 235 nt non-conserved region 5′ of the template in TER1 is highly divergent from both TER2 and other Brassicaceae, supporting the conclusion that this region is of recent origin. In *A. lyrata*, the 550 bp stretch 5′ of the RAD52 start is also highly divergent from TER2 and other Brassicaceae. However, this stretch in *A. lyrata* is highly similar to a segment of *A. thaliana* chromosome 2, but not TER1. In *A. arenosa* and *A. neglecta*, the region 5′ of the template domain is highly similar to TER2. Hence, the *A. thaliana* TER1 non-conserved region and the *A. lyrata* non-homologous stretch are of independent and recent origin. A well-documented genome rearrangement and chromosome number reduction occurred on the branch leading to *A. thaliana *(Lysak et al., 2009), which may explain the duplication of the TER locus, as well as the origin of the non-conserved regions of both TER1 and TER2. 

## DISCUSSION

The maintenance of telomeres is critical for genome integrity. In

Arabidopsis, the lack of functional telomerase leads to progressive telomere shortening, massive end-to-end chromosome fusions, and ultimately developmental and growth arrest in a terminal vegetative state (Riha et al., 2001). Arabidopsis is unique among all studied eukaryotes in encoding two TER subunits. TER1 plays the canonical role, forming an RNP complex with the telomerase reverse transcriptase TERT and acting to maintain telomere tracts on chromosome ends (Cifuentes-Rojas et al., 2011). In contrast, although TER2 can function as a template to direct telomere repeat addition *in vitro*, it is not required for telomere maintenance *in vivo* (Cifuentes-Rojas et al., 2011). Thus, the function of this RNA is unclear. Here we provide evidence that the duplication event producing the two *A. thaliana* TERs occurred within the last 13 million years (Beilstein et al., 2010), on the branch leading to *A. thaliana*. We cannot rule out the possibility that other Brassicaceae encode more than one TER, but such RNAs would have to arise from alternative loci since only a single region matching the TER1 and TER2 loci of *A. thaliana* was recovered from all sampled species. 

One of the most unexpected findings from this study is the marked divergence within the template domain of Brassicaceae TERs. The TER template domain is essential for telomerase function. Alterations to this domain can dramatically affect telomerase enzymology by reducing fidelity and processivity (Prescott and Blackburn, 1997; Rivera and Blackburn, 2004; Underwood et al., 2004). Even more significant, changes in the telomere repeat sequence incorporated onto chromosome ends have a cascading effect on the association of telomere capping proteins (Krauskopf and Blackburn, 1996), leading to severe genome instability and cellular senescence (Yu et al., 1990; Marusic et al., 1997). Thus, the telomere template domain is expected to be under strong purifying selection. Six of the putative TER loci we identified have canonical template domains identical to the corresponding regions in *A. thaliana* TER1 and TER2. Although the telomere sequence within the putative *Brassica rapa* TER includes an additional adenine, it retains an eight nucleotide stretch with perfect complementarity to the TTTAGGG telomere repeat, indicating that this locus could potentially encode a functional TER. In contrast, seven of the species we sampled exhibit extensive nucleotide substitution within the telomere sequence. Variations range from the insertion of additional nucleotides within the template domain in *C. hirsuta*, *C. rhomboidea*, and *A. arabicum*, to both insertion and deletion events in *C. lasiocarpa*. In these four cases, there is no contiguous stretch of seven nucleotides that can encode a TTTAGGG repeat. Remarkably, for three of the seven species, including *C. rubella* there is no telomere-like sequence at all within the TAD3 to RAD52 intergenic space. These loci do not encode alternative telomere repeat arrays, as canonical TTTAGGG repeats are found on the ends of genomic scaffolds in *C. rubella. *Therefore, some of the putative TER loci we identified cannot encode a functional TER, while for others the template domain may be compromised. 

The accessory protein POT1a assembles with TERT and TER1 into an enzymatically active telomerase RNP in *A. thaliana*. In particular, POT1a associates specifically with TER1, but not TER2, and acts to promote telomere maintenance (Surovtseva et al., 2007; Cifuentes-Rojas et al., 2011). POT1a binds TER1 within the 5′ non-conserved region (Cifuentes-Rojas et al., 2011). Consequently, we were surprised to discover a lack of sequence similarity between this region and the region 5′ to the template in our putative Brassicaceae TER loci. Given the level of divergence observed, it is possible that the POT1a–TER interaction is restricted to *A. thaliana*. Alternatively, the POT1a–TER binding site may be constituted by a conserved secondary structure element encoded by a highly variable nucleotide sequence. Finally, an alternative locus (or loci) may encode a TER that binds POT1a. Our current data cannot discriminate among these hypotheses.

Taken together, our findings indicate both a surprising amount of conservation and considerable evolution in the TAD3–RAD52 intergenic space. For some of the species we sampled, the locus we identified retains a telomere template domain and could potentially function as TER. If this is the case, Brassicaceae TERs are more conserved at the nucleotide level than TERs in other groups of organisms. For example, Brassicaceae TERs would share sequence identity of 90% in CR1 and CR2. While this conservation can be explained by the overlap of putative TERs with the RAD52 coding region, this level of conservation has not been observed in other groups where TER has been characterized (Chen et al., 2000; Dandjinou et al., 2004). However, it is possible that Brassicaceae TERs are similar to *A. thaliana* TER1 in that a significant portion of TER is encoded 5′ of the template domain. In this case, sequence similarity among the TERs would be greatly reduced. This level of variation would not be altogether surprising since long non-coding RNAs are known to evolve at an elevated rate compared with both microRNA and protein coding genes (He et al., 2011), although function is conserved (Pang et al., 2006).

Although all of the loci we identified retain CR1 and CR2, not all of the loci we identified appear to encode a functional TER since they lack the template domain. Thus, TER must be encoded at another locus in these genomes. This too is a surprising observation since the loss or degradation of the template domain is not correlated with phylogeny, indicating that the acquisition of alternative loci to encode TER is lineage-specific and much more frequent than has been demonstrated in vertebrates and yeast. Synteny of the *Saccharomyces sensu stricto* region that encodes TER is maintained over a 20 million year period (Naumov et al., 2000; Dandjinou et al., 2004), whereas in Brassicaceae the independent loss of the template domain in *A. lyrata*, *C. rubella*, and *L. draba* suggests new loci may have been co-opted over relatively short evolutionary intervals (~13 million years). In vertebrates, Chen et al. (2000) successfully employed a degenerate PCR approach to identify TER from 32 species across the vertebrate radiation spanning approximately 450 million years (Kumar and Hedges, 1998). Although they detected an elevated rate of evolution at the nucleotide level, eight domains were highly conserved. This stands in stark contrast to the situation in Brassicaceae; whole genome sequence is available for *A. lyrata* and *C. rubella*, yet we could not find any alternative loci with sequence similarity to either TER1 or TER2 that also contain a template domain. This observation raises the intriguing possibility that an alternative TER encoding locus in these species has little or no sequence similarity with other Brassicaceae TERs outside the template domain.

Finally, the evolution of TER in Brassicaceae could be dramatically different from yeast and vertebrates due to differences in selection acting on telomerase in plants. Arabidopsis can survive for up to ten organismal generations without functional telomerase (Riha et al., 2001), which would relax selection, possibly allowing for loss of one TER encoding locus and co-opting of another. Indeed the two Arabidopsis TERs may be a special case of relaxed selection, which initially allows the plant to tolerate both loci before they evolve more specialized functions (Innan and Kondrashov, 2010). Elucidation of the function of Arabidopsis TER2 and the other TER loci we identified will bring finer resolution to our evolving understanding of telomerase in plants and other eukaryotes.

## Conflict of Interest Statement:

The authors declare that the research was conducted in the absence of any commercial or financial relationships that could be construed as a potential conflict of interest.
